# Anaesthetic Considerations in a Patient with Pycnodysostosis undergoing Caesarean Delivery

**DOI:** 10.1155/2018/5675637

**Published:** 2018-11-13

**Authors:** Niklas S. Hansen, Karoline S. Dalgaard, Troels B. Jensen

**Affiliations:** Department of Anesthesia, Hospital Unit Vest, Herning, Denmark

## Abstract

Pycnodysostosis is a rare congenital disorder with several implications, which might complicate anesthesia. Patients are more prone to fractures and have an anticipated difficult airway. We report a case of a 34-year-old woman with pycnodysostosis who underwent elective caesarean delivery under epidural blockade.

## 1. Introduction

Pycnodysostosis is a very rare autosomal-recessive condition with a reported incidence of 1.7 per 1 million births [1]. The affected individuals are phenotypically characterized by short stature, usually less than 150 cm in height, brittle bones with a tendency to fracture after minor trauma, and spinal, cranial, and facial abnormalities. These abnormalities include frontoparietal bossing, thick calvaria, open fontanelles, hypoplastic paranasal sinuses, beaked nose, high arched palate, mandibular hypoplasia, and large protruding tongue [2]. These features might complicate the anaesthetic management. The craniofacial abnormalities may pose a challenge to endotracheal intubation via conventional direct laryngoscopy and the “sniffing” position, defined as neck flexion with upper cervical extension, may predispose to fracture of the cervical spine [3]. In this case report we describe the anaesthetic considerations and management of caesarean delivery in a patient with pycnodysostosis. A written patient consent was obtained.

## 2. Case Report

A 34-year-old primipara with pycnodysostosis was scheduled for an elective caesarean delivery in week 37 + 4 of pregnancy. The patient was 140 cm in height and weighted 60kg. She had no known allergies apart from nonsteroidal anti-inflammatory drugs and was not taking any prescribed medication. She had a history of multiple fractures, including 8 vertebral compression fractures in the thoracic and lumbar spine, but was otherwise healthy. Prior general anesthetics involving intubations had been without complications.

Prior to the caesarean delivery the patient was evaluated in a preoperative assessment by an experienced anesthesiologist.

The airway was assessed using the Simplified Airway Risk Assessment (SARI), which consist of 7 parameters: mouth opening, thyromental distance, Mallampati score, movement of the neck, ability to protrude the jaw, body weight, and a history of previous difficult intubation. A summed SARI score >3 indicates possible difficult intubation [4]. The patient had a SARI score of 3, due to inability to protrude her jaw (1 point) and a thyromental distance less than 6 cm (2 points). She had a modified Mallampati score of II. The overall dental status was good and she had no prior dental work done.

Physical examination of the spine revealed a slightly accentuated lumbar lordosis, which reduced with flexion. No scoliosis was detected.

Given her medical history, current pregnancy, and physical examination the initial plan was spinal anesthesia. Patient was also consented for general anesthesia. On the day of surgery the anesthesiologist responsible for the operation chose neuraxial anesthesia with an epidural approach. The patient was monitored with 3-lead electrocardiography, pulse oximetry and noninvasive blood pressure. The vital signs were all within normal ranges. Intravenous access was secured with an 18-gauge (G) cannula. Cefuroxime 1.5g and 1000 mL of Ringers-Acetate were administered. The epidural catheter was placed preoperative by an experienced anesthesiologist. Under strict aseptic conditions the epidural catheter was inserted at the L2-L3 level with an 18G Tuohy needle with the patient in the sitting position. The catheter was inserted via midline approach using the loss of resistance to saline technique. Loss of resistance was obtained at 5.5 cm, and the catheter was placed 3.5 cm into the epidural space. The procedure was uncomplicated. During the procedure the patient experienced a short period of paraesthesia in the left leg, which quickly resolved. There was negative aspiration for blood and cerebrospinal fluid. The catheter was tested with 2 mL of 2% lidocaine without onset of spinal anesthesia.

Epidural anesthesia was preformed with incremental doses of a solution consisting of 2% lidocaine with epinephrine 5*μ*g/mL, sodium bicarbonate 8.4mg/mL, and fentanyl 3.75*μ*g/mL. The epidural was dosed in aliquots with intermittent aspiration; a total of 6 mL was administered. The patient achieved satisfying level of analgesia above the Th4 level and the caesarean delivery was performed. It took 4 minutes from the beginning of the procedure to the delivery of a healthy boy. 10 international units of syntocinon were administered. Perioperative bleeding was estimated at 300 mL. The entire procedure took 26 minutes. The postoperative period was without complications.

## 3. Discussion

Pycnodysostosis also called Toulouse-Lautrec syndrome was first described in 1923 and defined by Mareteux and Lamy in 1962 [5]. The disease is equally distributed on both sexes [6]. The gene responsible for pycnodysostosis is located on chromosome 1q21. This causes a mutation in cathepsin K, a lysosomal cysteine protease, which is highly expressed in osteoclasts, and causes osteosclerosis due to decreased bone resorption [1]. The affected individuals are phenotypically characterized by short stature, usually below 150 cm, increased bone density, and tendency to fractures after minor trauma. Cranial and facial features include frontal and parietal bossing, beaked nose, prominent eyes with blue sclera, hypoplasia of the maxilla and mandible, open fontanels and sutures, thick calvaria, and hypoplastic paranasal sinuses [1, 2].

To the best of our knowledge a similar case of caesarean delivery in a patient with pycnodysostosis has not previously been described in the English literature. Failed intubation and other induction-related issues associated with general anesthesia continues to be the leading cause of maternal mortality and morbidity in caesarean delivery [7, 8]. But also extubation and the immediate postextubation phase have been identified as causes for maternal mortality associated with general anesthesia [9].

Our patient had facial dysmorphia including a large protruding tongue, hypoplasia and hypomobility of the mandible, and a SARI score of 3 and was 37-week pregnant, all of which contributes to potential difficult airway management. Difficulties with airway management in patients with pycnodysostosis have previously been described in the literature [10, 11]. Furthermore patients with pycnodysostosis are more prone to fractures, and manipulating the airway during intubation possesses a potential risk of trauma and fracture especially in the cervical spine.

As recommended by current consensus guidelines, the caesarean delivery was performed under neuraxial anesthesia [12]. In neuraxial anesthesia the final level of the block and the hemodynamic side effects are primarily dependent on patient height and the injected volume and concentration of local anaesthetic [13]. Since our patient was only 140 cm in height and scheduled for elective caesarean delivery, we choose to use an epidural catheter to slowly inject incremental doses of local anaesthetic until the desired effect.

Epidural was chosen over spinal blockade for several reasons: primarily to minimize the risks of inadvertent high block necessitating general anesthesia and endotracheal intubation; secondarily to reduce the risks of maternal hypotension, utero placental hypoperfusion, and fetal academia by reducing the speed of onset of sympathectomy.

Characteristic features of the spine in pycnodysostosis are scoliosis, kyphosis, and lumbar hyperlordosis [14]. Our patient had increased lumbar lordosis, decreased lumbar flexion, and old vertebral compression fractures. Although the lumbar spinous processes were easy palpable and placement of the epidural catheter turned out to be unproblematic, one might consider using neuraxial ultrasound as part of the preanaesthetic assessment in this type of patient.

The usage of neuraxial ultrasound for both spinal and epidural anesthesia can identify lumbar intervertebral levels more accurately than surface anatomical landmarks. Furthermore, it can predict the needle insertion depth to either the epidural or intrathecal space and it increases the success ratio for both epidural and spinal anesthesia in patients with normal anatomical landmarks and in patients with risk of difficult insertion [15].

In conclusion we present a patient with pycnodysostosis undergoing elective caesarean delivery under epidural anesthesia. We recommend a slowly titrated lumbar epidural neuraxial technique as the anesthesia of choice. As opposed to spinal anesthesia, the risk of high block and thus airway manipulation is reduced when using epidural anesthesia. Further, epidural anesthesia has a lower incidence of hypotension, utero placental hypoperfusion, and fetal academia compared to spinal anesthesia.
